# The association between dietary vitamin A intake and pancreatic cancer risk: a meta-analysis of 11 studies

**DOI:** 10.1042/BSR20160341

**Published:** 2016-11-22

**Authors:** Tao Zhang, Hongqiang Chen, Shiyong Qin, Minghai Wang, Xianming Wang, Xin Zhang, Fei Liu, Shuguang Zhang

**Affiliations:** * Department of General Surgery, Shandong Provincial Qianfoshan Hospital, Shandong University, Jinan, Shandong 250014, P.R. China

**Keywords:** meta-analysis, pancreatic cancer, vitamin A

## Abstract

Whether dietary vitamin A intake could reduce pancreatic cancer risk is still conflicting. We therefore conducted a meta-analysis to summarize the evidence from epidemiological studies. We searched the databases of PubMed and Web of Knowledge up to July 2016. Random model was used to combine study-specific relative risks (RR) and 95% confidence interval (CI). Publication bias was assessed by Egger regression asymmetry test and Begg's funnel plot. Eleven studies (10 case-control studies and 1 cohort study) involving 2705 pancreatic cancer cases were included in the present study. The RR (95% CI) of pancreatic cancer for highest category of vitamin A intake compared with lowest category was 0.839 (95% CI=0.712–0.988) with low heterogeneity detected (*I*^2^=17.8%, *P*_heterogeneity_=0.274). The relationships were also significant for studies designed by case-control [RR=0.808, 95% CI=0.690–0.947], as well as in European population [RR=0.821, 95% CI=0.693–0.972]. No evidence of publication bias was found. This meta-analysis demonstrated that dietary vitamin A intake might inversely associated with the risk of pancreatic cancer.

## INTRODUCTION

Pancreatic cancer is a common gastrointestinal cancer. Early diagnosis of pancreatic cancer is difficult because of the lack of clinical symptoms in the early stages, and the 5-year survival rate ranges from 4% to 6% or less [1,2]. Although the incidence and mortality of pancreatic cancer are remarkably high, few treatment options are effective. Therefore, primary prevention of pancreatic cancer is an important matter in the current society.

A recent study had reported that fruit and vegetable intake is associated inversely with the risk of pancreatic cancer [3]. Vitamin A is one of the most common antioxidants from fruits and vegetables, and it may exert chemopreventive effects [4]. Vitamin A could protect cells from oxidative DNA damage, thereby blocking carcinogenesis [5]. Furthermore, antioxidants have their effect on the inflammatory process, and chronic inflammation may play a role in pancreatic carcinogenesis [6]. To date, a number of epidemiologic studies have been published to explore the relationships between dietary vitamin A intake and pancreatic cancer risk. However, the results are not consistent. Therefore, the aim of the present study was to systematically examine whether dietary vitamin A intake could reduce the risk of pancreatic cancer.

## MATERIALS AND METHODS

### Search strategy and inclusion criteria

A comprehensive search was performed to identify all published studies on dietary vitamin A intake and pancreatic cancer risk. Searches of the PubMed and Web of Knowledge (up to July 2016) were conducted to identify eligible studies, with no language restriction. The key words used for the search strategy were ‘pancreatic’ and ‘cancer’ or ‘carcinoma’ and ‘vitamin A’ or ‘vitamins’ or ‘diet’ or ‘lifestyle’. Handsearching of reference lists of all relevant publications was finally done to identify more studies that could have been omitted from the databases’ search.

Studies were eligible for inclusion if the following predefined criteria were met: (1) using a case-control design or prospective design or cross-sectional design; (2) the association was between vitamin A and pancreatic cancer; (3) published as original articles; (4) contained the minimum information necessary to estimate the effects, i.e. odds ratio (OR) or relative ratio (RR) or hazard ratio (HR), and a corresponding measure of uncertainty, i.e. confidence interval (CI); and (5) the articles were included between 1980 and 2016. Otherwise, we excluded studies that had non-human subjects, duplicated data, those lack of OR or RR or HR, those conducted before 1980 and others that were mainly reviews, viewpoints or editorials. No language restrictions were applied.

### Data extraction and quality assessment

Two independent investigators (Tao Zhang and Hongqiang Chen) independently performed the abstract review and subsequent full text review. The key elements related to the study inclusion criteria were collected from each of the included studies. Extracted data will include OR or RR or HR of the outcomes, and their 95% CI, first author's name, study design, country of region, population size and statistical adjustment. The third reviewer (Shiyong Qin) or by contacting content experts were needed until the two reviewers reached a consensus when discrepancies appeared. The quality of each study was assessed using the established form first developed and applied by McShane et al. [7] and Hayes et al. [8]. Studies with scores ≥6 were considered high quality.

### Statistical analyses

RR and 95% CI were extracted from each study, which estimated the association for high compared with low vitamin A intake and pancreatic cancer risk. Random-effects model was used to combine the overall and subgroup results. Inconsistency was estimated using the *I*^2^ statistics; values of 25, 50 and 75% were considered low, moderate and high inconsistencies respectively [9]. Meta-regression and subgroup analyses (study design, geographic locations) were performed to assess the potentially important covariates that might exert substantial impact on between-study heterogeneity [10]. Sensitivity analysis was conducted to describe how robust the pooled estimator was to removal of individual studies [11]. Publication bias was evaluated using Egger regression asymmetry test [12] and Begg's funnel plot. STATA version 12.0 was used to analyse the results.

## RESULTS

### Flow of the included studies

**Figure 1:**
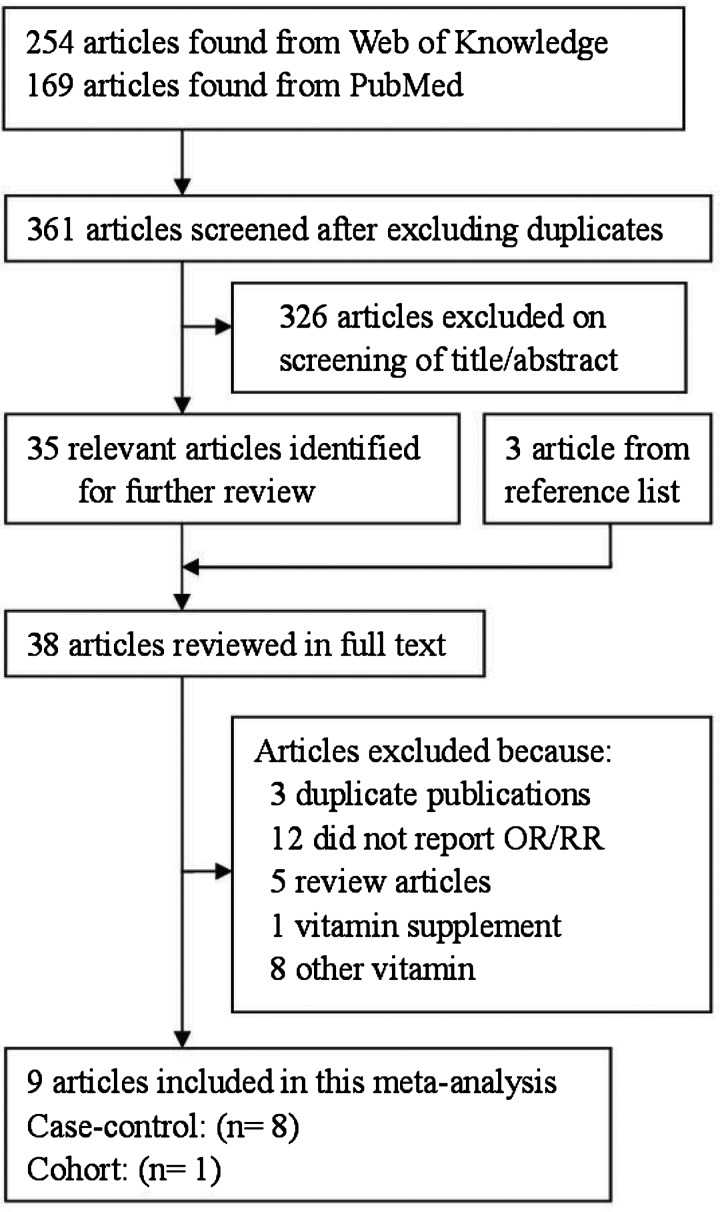
The flow diagram of screened, excluded and analyzed publications

Figure 1 showed a flow chart of studies that were suitable for this analysis. At the end, 11 studies [13–22] with 2705 cases were included in the study. Five studies were come from Europe, five from America and one from Asia. Ten studies were designed in case-control studies (six population-based case-control studies, four hospital-based case-control studies) and one in cohort. The characteristics of these included studies are presented in Table 1.

**Table 1:** Characteristics of the included studies

Study, year	Study design	Country	Age (years)	Cases	RR (95% CI) for highest compared with lowest category	Quality scores	Adjustment for covariates
Howe et al. (1990)	PBCC	Canada	35–79	249	1.12 (0.67–1.88)	7	Adjust for caloric and fibre intake, lifetime cigarette consumption
Olsen et al. (1991)	PBCC	United States	40–84	212	1.50 (0.70–3.00)	7	Adjusted for total energy, age, cigarette usage, alcohol consumption, respondent-reported history of diabetes mellitus and educational level
Zatonski et al. (1991)	PBCC	Poland	62.2	110	0.53 (0.20–1.45)	8	Adjust for cigarette lifetime consumption and calories
Kalapothaki et al. (1993)	HBCC	Greece	Not available	181	0.80 (0.65–0.99)	7	Adjust for age, gender, hospital, past residence, years of schooling, cigarette smoking, diabetes mellitus and energy intake
Stolzenberg–Solomon et al. (2002)	Cohort	Finland	50–69	163	1.21 (0.71–2.03)	8	Adjust for by the residual method and for age and years of smoking, energy-adjusted folate intake and energy-adjusted saturated fat intake
Lin et al. (2005)	PBCC	Japan	40–79	109	1.09 (0.62–1.92)	6	Adjust for age, pack-years of smoking and energy intake
Bravi et al. (2011)	HBCC	Italian	34–80	326	0.73 (0.44–1.19)	7	Adjusted for age, sex and centre, year of interview, education, tobacco smoking and history of diabetes, body mass index and total energy intake
Zablotska et al. (2011)	PBCC	United States	21–85	532	Male: 0.78 (0.42–1.50) Female: 0.78 (0.36–1.70)	8	Adjusted for energy intake by the residual method, body mass index, race, education, smoking, history of diabetes, physical activity and alcohol consumption
Jansen et al. (2013)	HBCC	United States	31–92	983	0.55 (0.37–0.81)	8	Adjusted for energy, smoking, BMI, age, sex and drinks of alcohol per week
Jeurnink et al. (2015)	PBCC	European	63.1	446	0.84 (0.50–1.40)	7	Adjusted for smoking status, duration and intensity of smoking, cotinine levels, waist circumference and diabetes status

### Vitamin A and pancreatic cancer

**Figure 2:**
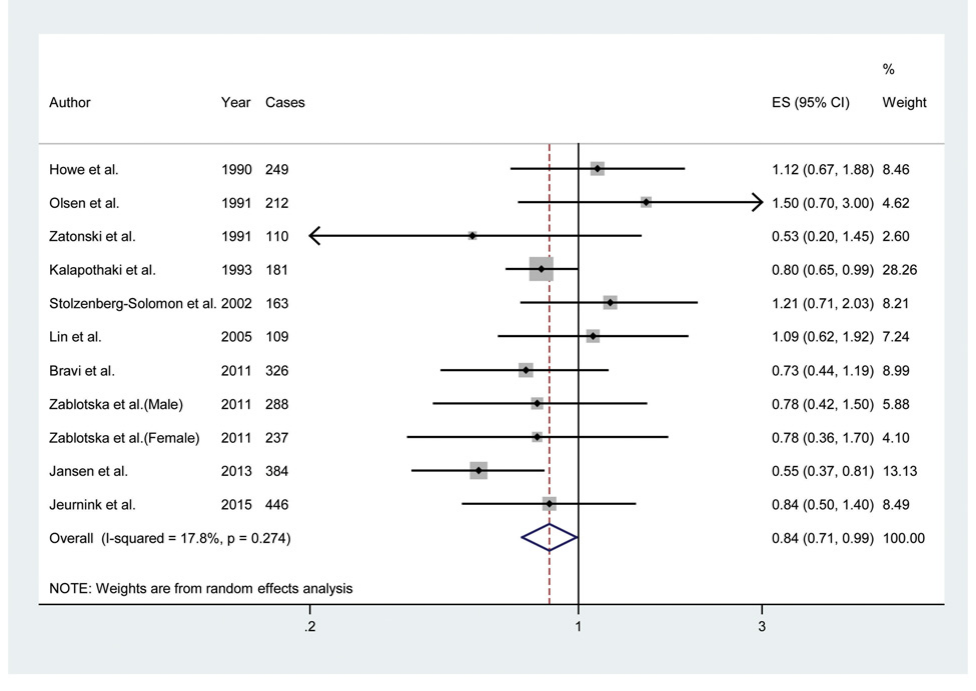
The forest plot between highest compared with lowest categories of dietary vitamin A intake and pancreatic cancer risk

Two individual studies reported an inverse association between dietary vitamin A intake and pancreatic cancer risk, whereas nine studies found no significant association between vitamin A intake and the risk of pancreatic cancer. Figure 2 presents the each RR with their 95% CI for all 11 studies assessing the association between vitamin A intake and risk of pancreatic cancer. The pooled RR of 0.839 (95% CI=0.712–0.988) shows that higher category of dietary vitamin A intake was associated with reduced risk of pancreatic cancer, with low heterogeneity detected (*I*^2^=17.8%, *P*_heterogeneity_=0.274).

### Meta-regression and subgroup analyses

As shown in Figure 2, low category of heterogeneity (*I*^2^=17.8%, *P*_heterogeneity_=0.274) was found in the pooled results. Thus, the univariate meta-regression was not performed.

Ten of 11 included articles were designed in case-control studies, so subgroup analysis for case-control studies was performed. Significant inverse association was found between dietary vitamin A intake and pancreatic cancer in case-control studies [summary RR=0.808, 95% CI=0.690–0.947]. Besides, we also extracted the detailed information for hospital based case-control studies (HBCC) or population bases case-control studies. And the significant associations were found in the hospital bases case-control studies [summary RR=0.745, 95% CI=0.632–0.878], but not in population based case-control studies (PBCC) [summary RR=0.985, 95% CI=0.753–1.288]. Studies were also stratified by geographic area, the RR were 0.821 (95% CI=0.693–0.972) for studies conducted in Europe, 0.852 (95% CI=0.588–1.233) for studies in America. There is only one study designed in cohort design and one study conducted in Japan. Thus, the subgroup analysis for them was not conducted. The detailed results are summarized in Table 2.

**Table 2:** Summary risk estimates of the association between dietary vitamin A intake and pancreatic cancer risk

Subgroups	Cases	Studies	RR (95% CI)	*I*^2^ (%)	*P*_heterogeneity_
All studies	2705	11	0.839 (0.712–0.988)	17.8	0.274
Study design					
Case-control	2542	10	0.808 (0.690–0.947)	10.0	0.351
PBCC	1205	6	0.985(0.753–1.288)	0.0	0.548
HBCC	1337	4	0.745(0.632–0.878)	0.0	0.398
Cohort	_	_	_	_	_
Geographic locations					
America	1370	5	0.852 (0.588–1.233)	50.1	0.091
Europe	1226	5	0.821 (0.693–0.972)	0.0	0.537

### Sensitivity analyses and publication bias

**Figure 3:**
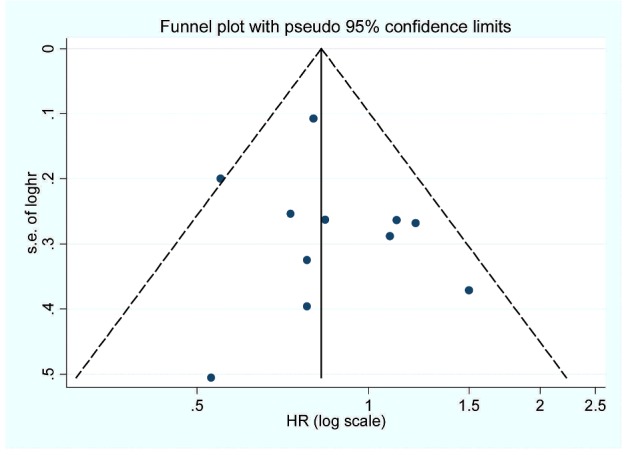
The funnel plot of the association between dietary vitamin A intake and pancreatic cancer risk

Sensitivity analysis showed that no individual study had excessive influence on the association of vitamin A intake and pancreatic cancer risk. Egger regression asymmetry test (*P*=0.483) and funnel plot (Figure 3) showed no evidence of significant publication bias between vitamin A intake and pancreatic cancer risk.

## DISCUSSION

The present study provided convincing evidence that dietary vitamin A intake is associated with a reduced risk of pancreatic cancer. This relationship between dietary vitamin A intake and pancreatic cancer risk was apparent and consistent in case-control studies and in the subgroup analysis of Europe populations.

A recent meta-analysis had been conducted to assess the association between vitamin E intake and the risk of pancreatic cancer [23]. The results in that meta-analysis found an inverse association between vitamin E intake and pancreatic cancer risk. For antioxidants, such as vitamins A and vitamin E, there are several plausible biological mechanisms by which they might prevent pancreatic cancer, including inactivating free radicals and reducing oxidative DNA damage, stimulating immune function [24]. Furthermore, dietary antioxidants have been shown in laboratory studies to enhance growth restriction of cancer cells in general and cancer cells in particular [25,26]. Vitamin A and vitamin E as antioxidant nutrients may influence this process due to their being free radical scavengers, suggesting that dietary antioxidants may play an important role in prevention.

As a meta-analysis of published studies, our findings showed some advantages. First, a highlight of the present study was that we found an inverse association between dietary vitamin A intake and the risk of pancreatic cancer with low between-study heterogeneity. Second, the present study included a large number of cases and participants; this may derive a more precise estimation of the relationship between vitamin A intake and pancreatic cancer risk. Third, no significant publication bias was detected in this meta-analysis. Fourth, the quality scores of each study are assessed with high quality. Fifth, between-study heterogeneity is common in the meta-analysis, and our study found no evidence of between-study heterogeneity in the pooled analysis and subgroup analysis.

There are some limitations in this meta-analysis should be concerned. First, our meta-analysis included 10 case-control studies and one cohort study. For the case-control studies, some recall or selection bias may be inherent in the original studies. Although prospective studies can allow a much greater possibility of reaching reasonable conclusions, the case-control study is an important method in aetiology research. Therefore, more original studies especially with prospective design are wanted in the future studies. Second, the association was only significant in the hospital-based case-controls, but not in the population-based case-controls. This may be caused by the little cases included in the population-based case-controls. Third, we did not do a dose-response analysis for vitamin A intake and the risk of pancreatic cancer because of the limited data in the reported articles. Further studies with detailed dose for each category are wanted to assess this association. Fourth, other unpublished literatures on relevant pharmaceutical websites were not searched and only studies in English were included, which may lead to a potential publication bias, although no significant publication bias was found by Egger's test and funnel plot. Finally, we only found significant association in the European populations, but not in the America and other populations. Therefore, more studies conducted in America and other areas are wanted to assess the relationship between dietary vitamin A intake and pancreatic cancer.

In summary, results from the present study showed clearly that dietary vitamin A intake can significantly reduce the risk of pancreatic cancer.

## Abbreviations

BMI: body mass index
CI: confidence interval
HBCC: hospital based case-control studies
HR: hazard ratio
OR: odds ratio
PBCC: population based case-control studies
RR: relative risks
